# Biased distance estimation in social anxiety disorder: A new avenue for understanding avoidance behavior

**DOI:** 10.1002/da.23086

**Published:** 2020-08-16

**Authors:** Nur Givon‐Benjio, Roni Oren‐Yagoda, Idan M. Aderka, Hadas Okon‐Singer

**Affiliations:** ^1^ Department of Psychology, School of Psychological Sciences University of Haifa Haifa Israel; ^2^ Department of Psychology, The Integrated Brain and Behavior Research Center University of Haifa Haifa Israel

**Keywords:** comfortable interpersonal distance, estimated interpersonal distance, estimation bias, social anxiety disorder

## Abstract

**Objectives:**

People regulate their interpersonal space appropriately to obtain a comfortable distance for interacting with others. Socially anxious individuals are especially prone to discomfort from and fear of physical closeness, leading them to prefer a greater interpersonal distance from others. Previous studies also indicate that fear can enhance the threat‐related elements of a threatening stimulus. For example, spider phobia is associated with estimating spiders as bigger and faster than they actually are. Nonetheless, it is still unclear whether the preference of those with social anxiety disorder (SAD) to maintain greater distance from others is associated with biased estimations of interpersonal distance.

**Materials and Methods:**

A total of 87 participants (44 clinically diagnosed with SAD and 43 control) performed validated computerized and ecological tasks in a real‐life setting while social space estimations and preferences were measured.

**Results:**

Participants with SAD felt comfortable when maintaining a greater distance from unfamiliar others compared to the control group and estimated unfamiliar others to be closer to them than they actually were. Moreover, the estimation bias predicted their preferred distance from strangers, indicating a strong association between estimation bias severity and actual approach‐avoidance behavior.

**Conclusion:**

Our findings indicate that distance estimation bias underlies avoidance behavior in SAD, suggesting the involvement of a new cognitive mechanism in personal space regulation.

## 1 INTRODUCTION

Social anxiety disorder (SAD) is the second most common anxiety disorder, with a lifetime prevalence of 12% (Bandelow & Michaelis, 2015). SAD is characterized by major fear in one or more social situations, and whenever possible individuals with SAD will choose to avoid their feared situation (Aderka et al., 2012; American Psychiatric Association, 2013; Rapee & Heimberg, 1997). Research studies have succeeded in identifying a few cognitive characteristics of SAD, including biases in attention (e.g., Lin, Qian, He, Wen, & Li, 2020; McGlade, Craske, & Nile, 2020), interpretation (Azoulay, Berger, Keshet, Niedenthal, & Gilboa‐Schechtman, 2020; Gutiérrez‐García, Fernández‐Martín, Del Líbano, & Calvo, 2019), memory (Amir & Bomyea, 2011; Yoon, Kutz, LeMoult, & Joormann, 2017), and perception (Brooks et al., 2008, Van de Cruys, Schouten, & Wagemans, 2013).

In recent years, research has begun to investigate the estimation bias of fear‐related stimuli as a new cognitive mechanism associated with anxiety disorders. For example, patients with spider phobia tend to estimate spiders as bigger and faster than they actually are (Leibovich, Cohen, & Henik, 2016; Shiban et al., 2016; Vasey et al., 2012; Witt & Sugovic, 2013). Similarly, heights appear higher to individuals with acrophobia (Stefanucci & Proffitt, 2009; Stefanucci & Storbeck, 2009; Teachman, Stefanucci, Clerkin, Cody, & Proffitt, 2008). Treatment was found to decrease overestimation of heights in acrophobia (Dreyer‐Oren, Clerkin, Edwards, Teachman, & Steinman, 2019) and of spider size in arachnophobia (Shiban et al., 2016), while manipulating this fear increased this bias in healthy participants (Clerkin, Cody, Stefanucci, Proffitt, & Teachman, 2009; Stefanucci, Proffitt, Clore, & Parekh, 2008), suggesting a strong association between participants' fear and their estimation bias. Nonetheless, it is worth noting that some studies have reported mixed or nuanced findings regarding estimation biases (e.g., Clerkin et al., 2009; Clerkin & Teachman, 2008; Hareli, Elkabetzn, & Hess, 2019). For example, Clerkin et al. (2009) failed to find differences in height overestimation when comparing individuals with high and low fear of heights. Furthermore, previous studies demonstrated that other nonvisual factors may also distort estimations of visual stimuli, such as extensive effort or fatigue (Bhalla & Proffitt, 1999; Witt, Proffitt, & Epstein, 2004), intentions (Witt et al., 2004), imagination (Clerkin et al., 2009), and expertise (Witt & Proffitt, 2005; Witt & Sugovic, 2010). Some of the mixed findings may be explained by the theoretical framework suggested by Proffitt (2006), according to which estimation biases emerge when one's actions have a potential cost (for more details, see Proffitt, 2006).

Previous studies have also indicated that SAD is characterized by avoidance of social interactions, manifested by a preference for maintaining a larger interpersonal distance, especially from strangers (Clark et al., 1995; Cohen & Shamay‐Tsoory, 2018; Perry, Rubinsten, Peled, & Shamay‐Tsoory, 2013). Physical closeness with social partner results in a considerable amount of stress among socially anxious individuals, in turn causing them to maintain greater distances from others (e.g., Perry et al., 2013; Rapee & Heimberg, 1997; Wieser, Pauli, Grosseibl, Molzow, & Mühlberger, 2010). Nonetheless, whether individuals with social anxiety only prefer to stay further away or whether they estimate the interpersonal distance in a distorted manner has yet to be examined. Recently, we (Givon‐Benjio & Okon‐Singer, 2020) conducted a pilot study on a nonclinical population to address this gap. We found that high levels of nonclinical social anxiety were associated with estimating the interpersonal distance from an unfamiliar partner as shorter. This distance estimation bias predicted the preferred distance, suggesting that the distance estimation bias plays a role in regulating interpersonal distance during social interaction. In the present study, our primary aim was to examine interpersonal distance estimations among individuals with a clinical diagnosis of SAD. We hypothesize that individuals with SAD estimate their interpersonal distance from strangers as shorter, so that the strangers appear to be closer in proximity. The second aim was to investigate modulation of personal space by the distance estimation bias. We hypothesize that this distance estimation bias is associated with distance preference, such that higher estimation bias will predict a preference for maintaining a greater distance from a social partner.

## 2 MATERIALS AND METHODS

### 2.1 Participants

The sample included 87 participants who participated in the experiment in return for payment: 43 individuals (22 men, M = 32, standard deviation [*SD*] = 11.9; 21 women, M = 25, *SD* = 5.1) who met the diagnostic criteria for SAD and 44 individuals who were not socially anxious (22 men, M = 27.5, *SD* = 4.3; 22 women, M = 27, *SD* = 8.9). All individuals underwent the Anxiety and Related Disorders Interview Schedule (ADIS; Brown & Barlow, 2014). The interviews were conducted by trained graduate students and supervised by a clinical psychologist. Levels of symptoms derived from measures of social anxiety were in line with the ADIS diagnosis. Specifically, the average LSAS for individuals in the SAD group was ∼80, similar to that reported in treatment studies for SAD and above the clinical cutoff of 60 (Rytwinski et al., 2009). The average LSAS for the control group was ∼25, which is also consistent with previous studies examining nonclinical samples (e.g., see Fresco, Coles, & Heimberg, 2001 for similar findings). Participants in the SAD group had a primary diagnosis of SAD (i.e., when other disorders are present, SAD is deemed the primary diagnosis), while participants in the control group had no diagnosis of SAD. Exclusion criteria for participation in all groups included: (a) frequent suicidal ideation (a score of 2 or more on item 9 of the Beck Depression Inventory II; Beck, Steer, & Brown, 1996); (b) past or present psychosis; (c) active psychotherapy or pharmacotherapy (except for stimulants for Attention Deficit Hyperactivity Disorder); (d) nonnative speakers of Hebrew; (e) neurological history; and (f) uncorrected vision.

During recruitment, participants were told that they were about to participate in a study that includes two computerized takes and an interview. When signing the consent form, participants knew that the study was associated with social anxiety and distance, but they did not know the tasks goal and the study hypotheses. Following the experiment, participants were fully debriefed on the tasks goals and hypotheses, in line with the ethical guidelines when using deception in a study.

### 2.2 Stimuli and design

The computerized and ecological tasks are detailed elsewhere (for detailed elaboration regarding the tasks, please see Givon‐Benjio & Okon‐Singer, 2020) and therefore we describe them only briefly here. The order of the computerized and the ecological tasks was counterbalanced across participants. The experimenter was not aware of the participants' group affiliation, yielding a blinded study.

#### 2.2.1 Computerized tasks

##### Descriptive phase

To verify that participants indeed imagined a specific person, before the computerized measurement they were asked to imagine one familiar friend and one familiar stranger (e.g., a neighbor they occasionally encounter but consider to be a stranger) and to describe each in a few words. Participants were encouraged to choose both the friend and the stranger freely and to describe as many details as possible, without any specific gender or other limitations. After completing the initial assignment, participants performed the computerized task, which was divided into two parts.

##### Computerized comfortable interpersonal distance task

**Figure 1:**
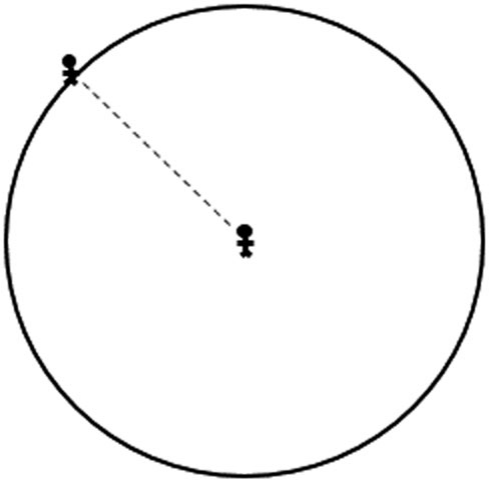
The computerized comfortable interpersonal distance task (Duke & Nowicki, 1972; Perry et al., 2013): in each trial, a circle image appeared and participants were asked to imagine that the figure in the center is themselves, and the figure on the perimeter is the friend\stranger. Then participants were asked to mark the dotted radius where they would start feeling uncomfortable with the proximity

This task is a measure of comfortable interpersonal distance (CID; Duke & Nowicki, 1972; Perry et al., 2013) with high reliability measures (for reviews, see Aiello, 1987; Hayduk, 1983). Participants view a circle with a figure at the center and another figure at the circle perimeter. Participants are then asked to imagine that they are standing at the center of a circular room and to imagine that the additional figure is either the “friend” or the “stranger” they chose in the descriptive phase (Figure 1). To clarify the identity of the additional figure, the word “friend” or “stranger” appeared 2,000 ms before the social scene was shown. A dotted radius appeared between the self and the friend/stranger, and the participants were instructed to mark the radius at the distance they would feel uncomfortable with the proximity. The radius was 90 mm, and the figure height was 12 mm. The additional figure, both the friend and the stranger, was placed at eight different locations along the perimeter. Each trial was repeated three times, resulting in 8 × 2 × 3 = 48 trials. Trials were presented in random order.

##### Computerized estimated interpersonal distance task

**Figure 2:**
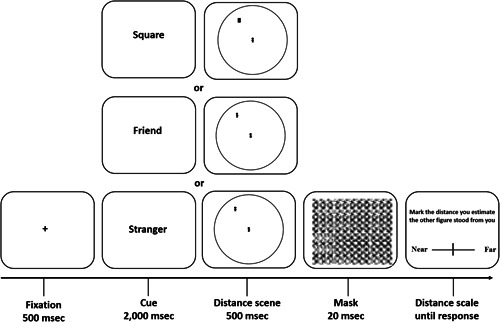
The computerized estimated interpersonal distance task timeline: participants viewed a fixation point, following by cue clarifying the additional fugue identity (i.e., stranger, friend, or square). Participants then were presented with the distance scene, and were asked to imagine that the figure at the center is themselves, and the additional figure is the specific friend or the stranger, they chosen at the descriptive phase, according to the cue. The scene was presented for 500 ms, followed by a short mask and a distance scale. Participants were asked to estimate the distance between themselves and the additional figure by marking the scale. We compared the differences in the distance judgments of the same distance, buy different identity

In this task, participants were shown a visual scene similar to the one in the cCID task. This time, however, the stranger/friend figure appeared inside the circle (and not along the perimeter) for 500 ms. After the image disappeared, a 20‐ms mask appeared, followed by a “near–far” continuous scale of 0–100, though the participants were not shown the scale's specific units. Participants were asked use the distance scale to estimate the distance between the additional figure and themselves. The additional figure was placed at 20 different locations, thus creating 20 different distances from the self. At each location the additional figure was identified either as a “friend” or as a “stranger.” This procedure allowed us to neutralize the subjective interpretation of the scale. Note that the fact that the visual stimuli were exactly the same enabled us to examine the distance estimation when all the subjective friend/stranger characteristics (e.g., hair color, height, and attractiveness) are controlled. Furthermore, a neutral stimulus (i.e., a square; Figure 2) was used for baseline nonemotional distance estimations. As in the case of the friend and the stranger figures, participants were instructed to report the distance between the square and themselves. In total, each of the additional figures (stranger/friend/square) was shown at 20 different locations that were repeated three times, resulting in 20 × 3 × 3 = 180 trials. Trials were presented in a random order.

#### 2.2.2 Ecological tasks

##### Ecological comfortable interpersonal distance task

**Figure 3:**
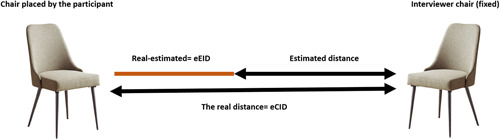
The ecological tasks: the real distance from the chair placed by the participants and the interviewer chair formed the ecological comfortable interpersonal distance (eCID). The estimated distance between the chairs reported by the participants was measured, and formed the estimated distance. To calculate the distance estimation bias, we compared the estimated and the real distance by subtracting one from the other, thus forming the ecological estimated interpersonal distance (eEID)

Participants were led to believe that they were about to be interviewed by an unfamiliar interviewer. The experimenter asked each participant to follow him to the interview room. Yet when the interview door opened, only one chair was in the room, while the additional chair (i.e., the participant's chair) was missing. The experimenter acted surprised, apologized, and asked the participant to place an additional chair inside the room for himself/herself while he (i.e., the experimenter) went to call the interviewer. The actual distance from the interviewer's chair at which the participant placed his/her chair inside the interview room represented the preferred distance from an unfamiliar other (Figure 3).

##### Ecological estimated interpersonal distance task

After the participant placed the chair inside the room (within ∼2–3 min), the experimenter came back, asked the participant to leave the room and go into the hallway, and closed the door. Then, the participant was asked to replicate the distance between the chairs in the interview room by placing two additional chairs at the same distance. The distance at which the participant placed the two chairs in the hallway represented the estimated interpersonal distance. The difference between the actual distance and the estimated distance represented the bias in estimating interpersonal distance (Figure 3). After participants completed the tasks, they were debriefed. As part of this debriefing, the experimenter first verified that the manipulation had worked by asking participants whether they believed an interview would take place. Note that all of the participants reported that the deception was successful. Specifically, participants reported that they believed an actual interview was about to take place when performing the tasks and did not suspect that the chair had been left outside the room intentionally.

Note that in both tasks, the estimation bias measurement was based on judgments made while the interpersonal distance was not visible to the participants. In this sense, this measurement approach differs from approaches used in previous investigations of estimation biases tied to clinical samples, in which the threatening stimulus was still present when the participants made the estimation (e.g., Stefanucci et al., 2008). In the current study, however, it proved difficult to prevent participants from using confounded visual aids to be more accurate, such as measuring the distance in the computerized task by putting their fingers on the computer screen or counting tiles in the ecological task. For this reason, the measurement approach used in the current study included spatial judgments while the scene was not visible to the participant.

## 3 DATA ANALYSIS

### 3.1 Computerized tasks

#### 3.1.1 Preferred distance (cCID)

The cCID score was calculated as the average preferred distance from the stranger minus the average preferred distance from the friend. A positive score represented a preference for a larger personal space from a stranger than from a friend. To examine the differences between the groups on the computerized measure of preferred distance, we computed an independent‐samples *t* test, with group (SAD/control) as the independent variable and cCID score as the dependent variable.

#### 3.1.2 Estimated distance (computerized estimated interpersonal distance task)

The computerized estimated interpersonal distance task (cEID) score was calculated by comparing the estimated distance from the friend and from the stranger at each specific location. Specifically, the estimated distance from the friend was subtracted from the estimated distance from the stranger, while the actual distance was the same. This procedure allowed us to examine the differences in the estimated distance from a visual stimulus that was the same but assigned a different identity. A higher cEID score represented the estimation bias, such that a stranger was estimated in closer proximity than a friend at the same distance. To examine the differences in distance estimation bias between groups, we calculated an independent‐samples *t* test, with group (SAD/control) as the independent variable and cEID score as the dependent variable. Furthermore, we calculated the estimated distance from a stranger compared to a neutral stimulus (i.e., the square) and examined the differences in the distance estimation bias between groups using independent‐samples *t* test.

### 3.2 Ecological tasks

#### 3.2.1 Preferred distance (ecological comfortable interpersonal distance task)

The ecological comfortable interpersonal distance task (eCID) score was calculated as the distance (in cm) between the chair that the participant placed in the room and the interviewer chair already in the room. A higher ecological estimated interpersonal distance (eEID) score represented a preference for a greater interpersonal distance from an unfamiliar interviewer. An independent‐samples *t* test was calculated to examine the differences in preferred distance between the groups on the ecological measure, with group (SAD/control) as the independent variable and eCID score as the dependent variable.

#### 3.2.2 Estimated distance (eEID)

The eEID score was calculated by subtracting the estimated distance (i.e., the preferred distance) between the chairs from the actual distance. A higher eEID score represented biased estimation of personal space, such that the unfamiliar interviewer was estimated to be closer. An independent‐samples *t* test was calculated to examine the differences in distance estimation bias between the groups on the ecological measure, with group (SAD/SAD posttreatment/control) as the independent variable and eEID score as the dependent variable.

### 3.3 Using the distance estimation bias to predict the preferred distance

A Pearson correlation was calculated to examine the correlation between the estimation bias and the preferred distance so as to predict the distance preference from the distance estimation. This Pearson correlation was conducted separately for each of the tasks (computerized vs. ecological) and for each of the groups (SAD, control), resulting in six Pearson analyses.

Outliers were defined as 3.3 SDs above or below the group average (see Osborne & Overbay, 2004). There were three outliers, which were corrected using winsorization (Ghosh & Vogt, 2012).

## 4 RESULTS

### 4.1 Computerized tasks

#### 4.1.1 cCID task

**Figure 4:**
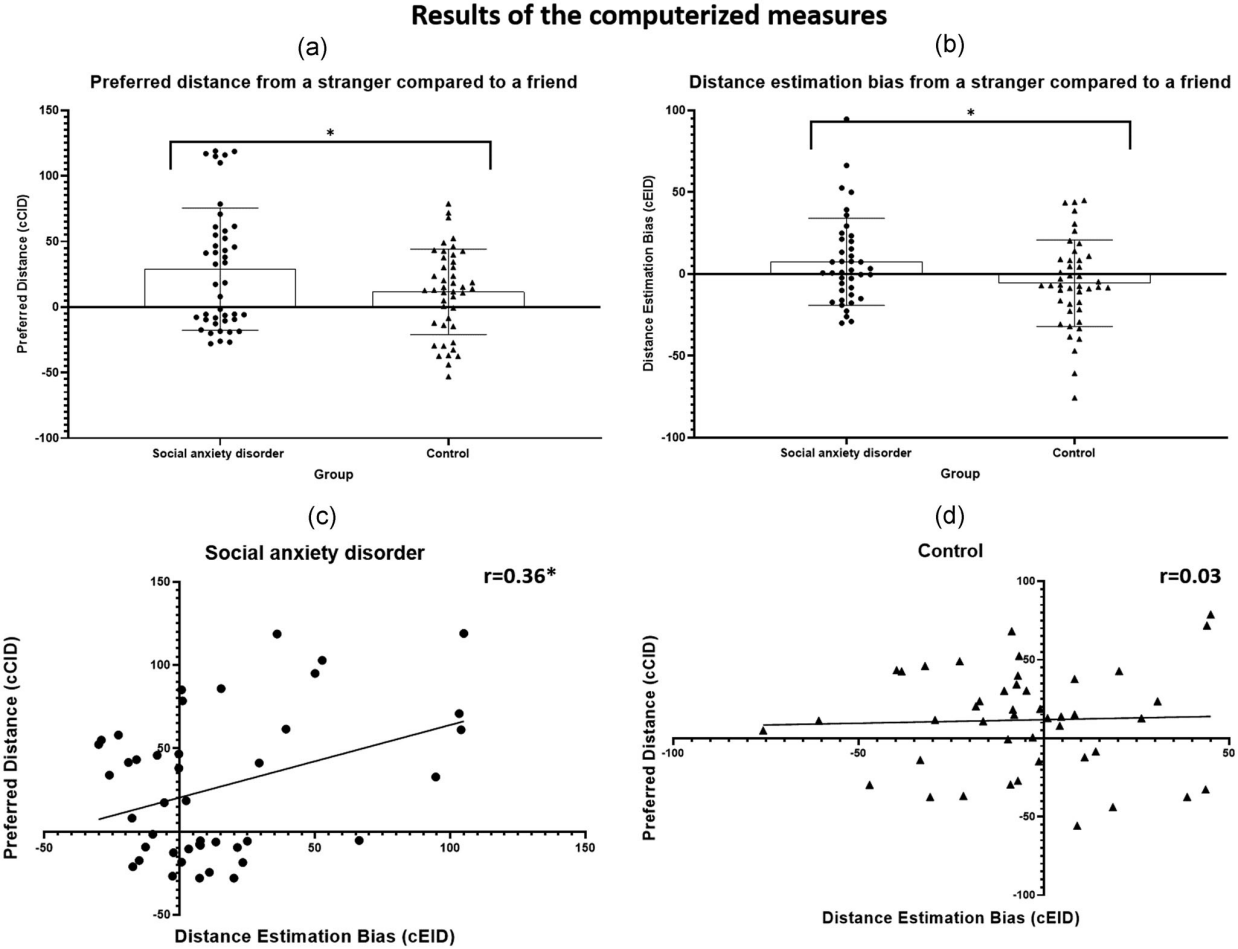
The results of the computerized tasks. (a) Preferred distance: the social anxiety disorder (SAD) group demonstrated a preference to maintain a greater interpersonal distance from the stranger compared to the controls (*t*(75) = 2, Cohen's *d* = 0.4, *p* = .048). (b) Estimated distance: the SAD group demonstrated higher distance estimation bias compared to the controls (t(85) = 2.95, Cohen's *d* = 0.63, *p* = .004). Specifically, the SAD participants estimated the distance from a stranger as shorter, such that the stranger was estimated in closer proximity. (c) SAD: a significant correlation was found (*r* = .36, *r* ^2^ = 13%, *p* = .02). Specifically, participants how underestimated the interpersonal distance also preferred to maintain a greater distance from the stranger. (d) Control: no significant correlation was found (*r* = .03, *r* ^2^ = 0, n.s)

An independent‐samples *t* test was used to examine the difference in preferred distance between the groups. A significant difference in cCID score emerged for the SAD group (M = 29, *SD* = 46.59) compared to the control group (M = 11.6, *SD* = 32.57); (*t*(75)= 2, Cohen's *d* = 0.4, *p* = .048; see Figure 4a). Specifically, on the computerized measure participants with SAD preferred to maintain a greater interpersonal distance from the stranger than did controls.

#### 4.1.2 cEID task

An independent‐samples *t* test was used to examine the difference in estimated distance between the groups. A significant difference in cEID score emerged for the SAD group (M = 14, *SD* = 35.74) compared to the control group (M = −5.6, *SD* = 26.48); (t(85)= 2.95, Cohen's *d* = 0.63, *p* = .004; see Figure 4b), such that participants with SAD exhibited a higher distance estimation bias than controls. Specifically, participants with SAD estimated the stranger to be closer than the friend, even though the friend was positioned at the same physical distance from them.

Furthermore, no significant group difference in social anxiety emerged between the conditions of estimated distance from a friend and from a square (t(85)= 0.5, Cohen's *d* = 0.1, *p* = .43), such that participants estimated the distance from a square as similar to the distance from a friend. This result suggests that in the stranger‐friend comparison, the effect may have emerged due to estimating the stranger as closer and not the friend as farther away. Nevertheless, we expected that the comparison between the estimated distance from a stranger compared to from a square would be significant, such that participants would underestimate the distance from a stranger when compared to the distance from a neutral stimulus (the square). Although there was a trend in the expected direction, this comparison did not reach significance (t(85)= 0.77, Cohen's *d* = 0.16, *p* = .43).

#### 4.1.3 Correlation between cCID and cEID scores

To better understand the extent to which the estimation bias predicted the distance participants preferred to maintain from a stranger, we examined the correlation between the estimated distance (cEID score) and the preferred distance (cCID score) in each of the groups separately. We found a positive correlation between the cEID score and the cCID score in the SAD group (*r* = 0.36, *r* ^2^ = 13%, *p* = .02; Figure 4c), but did not find a significant correlation in the control group (*r* = .03, *r* ^2^ = 0, *p* = .8; Figure 4d). In other words, the higher their estimation bias, the greater the distance that participants with SAD preferred to maintain from a stranger.

### 4.2 Ecological tasks

#### 4.2.1 eCID task

**Figure 5:**
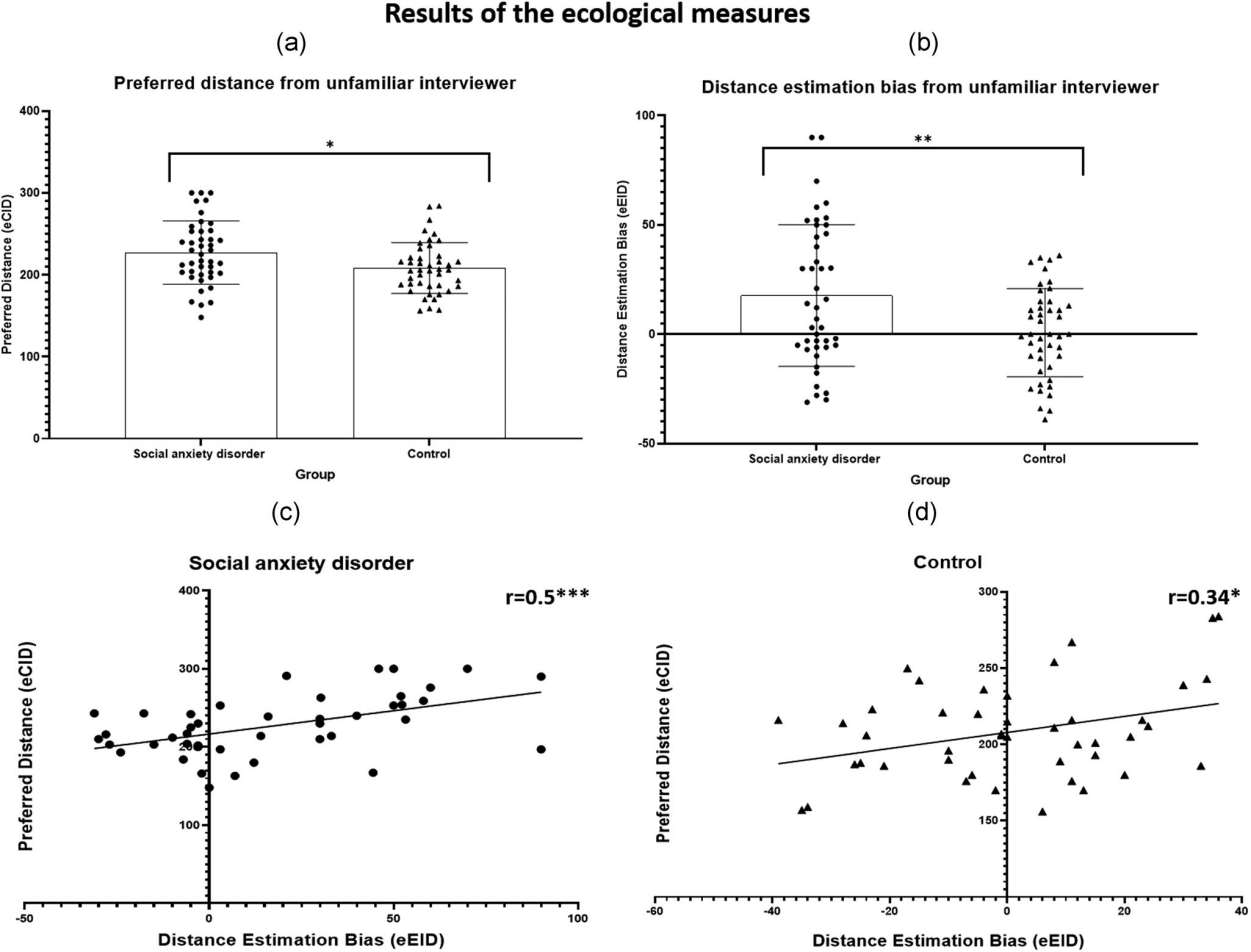
The results of the ecological tasks. (a) Preferred distance: the social anxiety disorder (SAD) group demonstrated a preference for greater interpersonal distance from the stranger compared to the controls (t(85) = 2.51, Cohen's *d* = 0.5, *p* = .01). (b) Estimated distance: the SAD group demonstrated higher distance estimation bias compared to the controls (t(70) = 2.93, Cohen's *d* = 0.6, *p* = .004). Specifically, the SAD participants estimated the distance from a stranger as shorter, such that the stranger was estimated in closer proximity. (c) SAD: a significant correlation was found (*r* = .5, *r* ^2^ = 25%, *p* = .001). Specifically, participants how underestimated the interpersonal distance also preferred to maintain a greater distance from the stranger. (d) Control: a significant correlation was found (*r* = .34, *r* ^2^ = 11%, *p* = .023)

An independent‐samples *t* test was conducted to examine the difference in preferred distance between the groups. A significant difference in the eCID score emerged in the SAD group (M = 227, *SD* = 38.6) compared to the control group (M = 208, *SD* = 31) (t(85) = 2.51, Cohen's *d* = 0.5, *p* = .01; see Figure 5a). Specifically, on the ecological measure, participants with SAD preferred to maintain a greater distance from the stranger than did the control group members.

#### 4.2.2 eEID task

An independent‐samples *t* test was conducted to examine the difference in estimated distance between the groups. A significant difference in the eEID score emerged in the SAD group (M = 17.7, *SD* = 32.4) compared to the control group (M = 0.7, *SD* = 20.1) group (t(70) = 2.93, Cohen's *d* = 0.6, *p* = .004; see Figure 5b). Specifically, participants with SAD estimated the distance from the unfamiliar interviewer as shorter than the actual distance. The task split‐half reliability was good (Spearman–Brown coefficient = .838).

#### 4.2.3 Correlation between eCID and eEID scores

To determine whether preferred distance can be predicted by distance estimations, we examined the correlation between the estimated distance (eEID score) and the preferred distance (eCID score) in each of the groups separately. We found a positive correlation between the cEID score and the cCID score in the SAD group (*r* = .5, *r* ^2^ = 25%, *p* = .001; Figure 5c), such that the higher their estimation bias, the greater the distance participants with SAD preferred to maintain from a stranger. A significant correlation was also found in the control group (*r* = .34, *r* ^2^ = 11%, *p* = .023; Figure 5d).

## 5 DISCUSSION

In this study, we investigated biases in interpersonal distance estimation among individuals with SAD as well as the association between the biased estimation and personal space regulation. The results show that SAD is associated with distorted estimation of interpersonal distance, such that unfamiliar others are estimated to be closer than they actually are. This bias, in turn, is associated with the proximity with which an individual feels comfortable. Our results also indicate that the estimation bias is specific toward strangers and not toward friends or a neutral stimulus, suggesting that this bias is fear related. To the best of our knowledge, this is the first demonstration of distance estimation bias in SAD, and as such, the findings have important implications for understanding abnormalities in personal distance regulation and avoidance.

Nevertheless, it is important to note that in the computerized task, the stranger‐square comparison did not reach significance. That is, participants estimated the distance from the stranger as similar to the distance from the square. Therefore, it is possible that the estimation bias that was found in the stranger‐friend comparison was due to the friend (i.e., participants overestimated the distance from the friend) and not due to the stranger (i.e., participants underestimated the distance from the stranger). The fact that we found the estimation bias in the ecological task (which included a stranger) as well as in a previous study (Givon‐Benjio & Okon‐Singer, 2020) provides support for the estimation bias. However, we cannot completely rule out alternative explanations for the null findings, and future studies may be warranted to further examine this issue.

Our findings can be interpreted in the context of cognitive‐behavioral models of SAD (Clark et al., 1995; Hofmann, 2007; Rapee & Heimberg, 1997). If individuals with SAD estimate the distance from strangers as shorter and prefer to maintain larger interpersonal distances from others, they consequently will keep their distance physically. This increased distance can be understood as a safety behavior intended to reduce anxiety. Such safety behaviors have been found to impair social performance (Rowa et al., 2015), increase momentary anxiety (see Piccirillo, Dryman, & Heimberg, 2016 for a review), and even lead to interpersonal rejection, which further fuels future anxiety (Alden & Taylor, 2010; Plasencia, Taylor, & Alden, 2016). Thus, biased estimation of interpersonal distance may play an important role in predisposing individuals to a process of maintaining/exacerbating the disorder.

An alternative perspective on our findings can be derived from interpersonal models of SAD (e.g., Alden & Taylor, 2010). According to such models, individuals with SAD adopt self‐protective behaviors in interpersonal interactions (e.g., keeping one's distance). These behaviors may inadvertently lead to a host of negative reactions from others, thus affirming the negative perceptions held by individuals with SAD. For instance, self‐protective behaviors such as maintaining physical distance may lead to perceptions of dissimilarity from interaction partners (Voncken, Alden, Bögels, & Roelofs, 2008) or to partners experiencing the interaction as less smooth, coordinated, and enjoyable (Heerey & Kring, 2007). These, in turn, may lead to interpersonal rejection or to diminished desire for future interaction (Alden & Taylor, 2010). Thus, biased estimation of social distance as well as preferences for maintaining greater social distance may play a role in deleterious maintenance processes in SAD. Considering that biased estimation of social distance and preferences for maintaining greater social distance may play a role in maintaining SAD, in the therapy session clinicians may choose to inquire about clients' preferences for distance. Thus, instead of assuming that norms of interpersonal distance within a certain context or culture apply to individuals with SAD, facilitating an open conversation about what distance the client feels comfortable with may help foster an atmosphere of collaboration as well as convey to clients that the therapist values their input and can make adjustments to make them feel more comfortable. This in turn may contribute to a strong therapeutic alliance, which has been found to predict treatment outcome across therapeutic orientations and disorders (see Martin, Garske, & Davis, 2000 for a review).

In addition, our findings suggest that developing interventions to target biases in distance estimation may be helpful for individuals with SAD and can potentially enhance and augment existing treatments. Identifying cognitive biases such as attentional biases and interpretation biases in SAD has led to the development of such interventions. Moreover, these interventions have been empirically examined and found to reduce social anxiety (Beard & Amir, 2008; Heeren, Mogoase, Philippot, & McNally, 2015; for attention and interpretation biases, respectively). Thus, future studies can focus on the development and examination of interventions that target distance estimation, which have the potential to reduce social anxiety and enhance existing treatments.

The present research has several limitations. First, in the computerized tasks, we were unable to confirm whether participants successfully imagined the friend and the stranger when asked. Previous studies, however, have shown that participants' responses were affected even when the threatening stimuli were task‐irrelevant (Hodsoll, Viding, & Lavie, 2011; Okon‐Singer, 2018) and even when the threatening stimulus was presented subliminally without their conscious awareness (Siegel, Selvaggi, Sims, & Rinck, 2019). Importantly, Clerkin et al. (2009) demonstrated that when participants were asked to imagine themselves falling, the overestimation of the height increased, suggesting that imagination can affect participants' reaction. Hence, it is reasonable to assume that the participants' reactions were affected by the identity of the figures.

The second limitation is that the specific cognitive function underlying this bias is unclear. On one hand, it is possible that this bias is due to distorted perception processes. Supporting this notion are studies showing that fear and perceived danger influence the perception of altitude, size, and slant (Stefanucci & Proffitt, 2009; Stefanucci et al., 2008; Stefanucci, Gagnon, Tompkins, & Bullock, 2012; Teachman et al., 2008). In those studies, participants estimated the distance while viewing it, while in both our tasks the scene was not visible to the participants at the time of the judgment. Therefore, the estimation bias may have been due to memory dysfunction. In line with this notion, studies have reported deficiencies in working memory in participants with SAD compared to controls (Adami, Mahmoud, & Nazari, 2019). Nonetheless, studies have also reported that SAD is associated with enhanced working memory of their feared stimulus compared to positive and neutral stimuli (Amir & Bomyea, 2011; Yoon et al., 2017), suggesting that the SAD group should have been more accurate in their judgments of distance from strangers than from friends and from the square. Furthermore, previous studies have demonstrated estimation bias when the threatening stimulus was present at the time of the judgment (Stefanucci & Proffitt, 2009; Stefanucci et al., 2008; Vasey et al., 2012; Shiban et al., 2016; Witt & Sugovic, 2013). This finding supports the claim that memory is not the cognitive mechanism underlying the estimation bias, or at least not the only mechanism. Nonetheless, we believe that for the sake of future research it is important to replicate our results while controlling for memory.

Another possibility is that the interpersonal distance was underestimated due to attentional bias. Supporting this notion are studies indicating that SAD is characterized by self‐focused attention. That is, socially anxious individuals tend to focus their attention on internally generated information (such as their body state, their thoughts, and emotions) at the expense of focusing on relevant external information (Clark et al., 1995; for a recent review, see Schwarzer & Wicklund, 2015). Therefore, individuals with SAD may estimate interpersonal distance incorrectly because they do not allocate sufficient attention resources to the social partner. In light of these findings, future studies should examine the cognitive mechanism underlying the distance estimation bias, including though not limited to perception, memory, and attention.

The combined results of our study represent the first demonstration of biased estimation of interpersonal distance in SAD, suggesting that the estimation bias plays a role in regulating interpersonal distance during social interaction. These innovative findings have important implications for the understanding of avoidance behavior.

## CONFLICT OF INTERESTS

The authors declare that there are no conflict of interests.

## Data Availability

The data that support the findings of this study are available from the corresponding author, N. Givon‐Benjio, upon reasonable request.
